# PD66 Scalability Of Integral, Transversal, And Multidisciplinary Management Of Aortic Stenosis In Catalan Hospitals

**DOI:** 10.1017/S0266462324003234

**Published:** 2025-01-07

**Authors:** Franka Van Scheepen, Carla Fernandez-Barcelo, Bàrbara Vidal Hagemeijer, Ismail Abbas, Marta Sitges Carreño, Laura Sampietro-Colom

## Abstract

**Introduction:**

A program for integral, transversal, and multidisciplinary management of aortic stenosis (MITMEVA) was implemented at the Clinic Barcelona University Hospital to provide adequate and personalized treatment for patients with aortic stenosis (AS). MITMEVA includes 11 actions in the AS care pathway. This study aimed to test the scalability of MITMEVA by gathering the perspectives of relevant stakeholders in two other Catalan hospitals.

**Methods:**

A mixed method study design was used to collect and analyze the views of relevant stakeholders on the scalability of the MITMEVA project. Qualitative and quantitative methods were used to answer the research question. All participants were interviewed individually using a semi-structured interview guide. The interviews were transcribed and analyzed by performing a content analysis with Atlas.ti software. Quantitative data were gathered and analyzed by adjusting the Intervention Scalability Assessment Tool (ISAT).

**Results:**

Nine participants were interviewed from two tertiary hospitals in Catalonia and eight ISAT questionnaires were completed. From the content analysis, 11 of the 15 themes identified related to actions implemented in the MITMEVA program. The results pointed toward a positive view of implementation of the MITMEVA program in general, showing a good scalability for all the ISAT domains. On a scale of zero to three, most domains were scored two or above.

**Conclusions:**

The qualitative and quantitative results indicated that the MITMEVA program has potential for scaling up to other Catalan hospitals to improve the management of AS. Generally, stakeholders from the two participating hospitals were positive about the project. However, small adjustments will be needed to account for the culture and organizational characteristics of the hospitals when scaling up the MITMEVA program.

